# Supplementary material for: BoReMi: Bokeh-based jupyter-interface for registering spatio-molecular data to related microscopy images
Source: PLoS Comput Biol. 2024 Oct 7;20(10):e1012504. doi: 10.1371/journal.pcbi.1012504 (PMC11486395; doi:10.1371/journal.pcbi.1012504)
Supplement: Attachment — Submitted filename: PbP_forSubmission.pdf [file pcbi.1012504.s001.pdf]

Dear Dr. Gokce, Dear Dr. de Souza,

Thank you for taking the time to review our manuscript "*BoReMi: Bokeh-based jupyter-interface for Registering spatio-molecular data to related Mi-croscopy images*" and test BoReMi. Your comments have helped us to significantly improve BoReMi itself as well as its presentation in the manuscript.

**The key improvements of this revision include:**

- **New BoReMi functionalities:** BoReMi now includes the possibility of non-linear (elastic) adjustments, colored data point display, and undo/redo/reset functionalities. The new version of BoReMi (v0.2.0) is available for interactive testing in Binder through github (https://github.com/jaspreetishar/BoReMi).
- **Improved documentation:** Detailed installation and setup instructions are now available in the README as well as the notebook itself.
- **Demonstration of multiple use-cases:** We now include multiple data types (MERFISH and Slide-seq) and tissue types (human breast cancer metastasis and mouse brain) and demonstrate registration of multiple serial sections, single-cell matching in a DAPI image, non-linear adjustment, and registration to not-perfectly matching data base images.
- **Benchmarking and comparison:** We now quantify the goodness of alignment by calculating spatial correlations of gene expression between serial sections as well as % overlap between data and HE image. And we compare BoReMi to STalign, demonstrating a use-case where automated alignment leads to over-distortion.
- **Manuscript text and figures:** We have significantly revised and restructured the text for more streamlined reading and clarity. Both original figures have been revised and 3 new figures have been added.

Please find attached a detailed point-by-point reply to your comments. With the revisions outlined above and detailed in the point-by-point response below, we are confident that BoReMi and our manuscript will be of great interest to the readership of *PLOS Computational Biology* and the broader spatial omics community.

Sincerely,

Johanna Klughammer (on behalf of all co-authors)

**Point-by-point response to reviewer comments**

**Reviewer #1:** Ishar et al. have developed an innovative python-based, interactive visual tool, BoReMi, for the manual alignment of spatial molecular data to images through rigid transformations. This tool emerges as a particularly valuable resource amid the growing adoption of spatial molecular profiling technologies, offering an integrative and interactive solution that stands out in the current landscape of similar software. BoReMi's functionality is both timely and highly beneficial, manuscript written clearly and code is clear. I recommend its publication after minor edits.
My comments below, largely reflective of enhancements for a potential version 2, are intended to further distinguish BoReMi from competing methodologies. Additionally, I suggest several improvements to the manuscript for a more impactful presentation of BoReMi's capabilities:
Thank you for your helpful comments and suggestions and your appreciation of BoReMi.

1- Non-linear Transformations: The tool currently supports rigid transformations, showcasing exceptional performance with large imaging files. This feature is invaluable for initial alignment tasks. However, for comprehensive utility, it would be beneficial if the discussion section could recommend non-linear alignment tools that complement BoReMi for subsequent alignment steps.

We agree that non-linear transformations are important and have now implemented this feature as an elastic drag-and-drop movement of individual data points and their neighbors within a certain user-defined threshold. Since this feature is very powerful and can severely bias downstream analysis, we release it as “beta” and for now we refrain from adjusting biological structures. Instead, we demonstrate this feature by correcting a slight shift in the data possibly due to a technical artifact such as a tear (Figure 3a). Such small-scale technical artifact repair is for now the suggested use-case of this feature although it has the potential to also adjust larger distortions. This new feature is also available for beta-testing in the BoReMi Binder.

2- Streamlining the Results Section: The middle part of the Results section, which details a specific analysis process, could be condensed or moved to supplementary materials or an online guide. This adjustment would allow the main text to more effectively highlight BoReMi's versatile features and their applicability across various scenarios.

As suggested, we now moved the detailed description of the alignment process to the “Design and Implementation” section and now present different scenarios and benchmarking results in the results section.

3- Visualization Capabilities: It would be valuable if the authors could discuss whether BoReMi can display cluster assignments in different colors and how it utilizes the inherent molecular

information of spatial transcriptomics data. Additionally, the ability to handle and visually represent continuous molecular expression data would significantly enhance BoReMi. These points can be added as how to or in discussion.

We managed to implement the multi-color display of data points. However, the display of continuous molecular expression data in its current state would considerably reduce BoReMi's efficiency and responsiveness. Therefore, we can only mention this useful feature in the "Future Directions" section.

4- Computational Performance: The manuscript should detail the computational requirements of BoReMi, especially how it scales with the size of the molecular data and image database. Given the substantial size of Merscope files, ensuring that user has the right tools to run BoReMi.

We have now added a section on computational requirements to the manuscript and github: "BoReMi has modest resource requirements: On a Surface Pro 7 tablet (Intel Core i5-1035G4, 16GB memory) it runs smoothly and opens the Merfish mouse brain dataset with 83k cells (subsampled to 10k cells for visualization) and images with 100 MP in about two minutes for PNG images and in about 1 minute for JPEG images. Downsampling the number of displayed data points to 10k, while all data points are being processed, ensures lag-free interaction even with modest computational resources."

Minor Points:

- The manuscript hints at the potential for processing multiple spatio-molecular data sets simultaneously but does not delve into this feature. Clarification on the concurrent visualization and independent manipulation of different data sets would be beneficial.

We have now added a show-case for the processing of multiple sections and display the results in Figure 4. Tabs allow efficient switching between sections as well as individual manipulations to achieve alignment.

- Introducing a record log of all performed transformations would aid reproducibility and enable users to replicate alignments with other tools.

BoReMi now allows the user to download the final alignment parameters as well as a record log (of the linear transformations). Non-linear transformations are non-deterministic and not directly logged.

- Further explanation on whether users can select which data set serves as the reference during alignment and the possibility of applying transformations in reverse would enhance BoReMi's flexibility.

We edited the text to clarify and emphasize that the image serves as reference by specifying the process as “data-to-image registration” and we implemented a “undo-redo” functionality.

- In its current form, we will likely use BoReMi as the first step to check alignment pairs before applying elastic like tools. These suggestions aim to refine both BoReMi's functionality and its presentation which are already at a very high standard.

Again, thank you for your helpful suggestions which have significantly improved BoReMi. We hope that the new features and especially the non-linear (elastic) manipulation will benefit your work.

**Reviewer #2:** The manuscript from Ishar, Tam and Klughammer describes the tool BoReMi for aligning spatial molecular data (i.e., spatial distributions of transcripts or proteins) to imaging data such as from Haematoxylin & Eosin stains of tissue. Such alignment is key if one is to make biological sense of molecular data, since these data typically lack information about tissue structure. Further, more and more such spatio-molecular datasets are likely to be generated and analysed in the next years. Tools in this sphere are therefore important and timely, and as such this paper is potentially of wide interest. BoReMi is easy to use, doesn't need coding experience, and has a nice interactive demo – these are all strengths.

Thank you for recognizing BoReMi's potential and your detailed feedback as well as valuable suggestions.

In its present form however, the manuscript is very minimalist. To us it reads as more of an announcement of the tool than as a research paper. There are several points we think should be addressed before the paper can be published in PLoS Computational Biology.

We have now significantly expanded our manuscript with 3 completely new figures and restructured the text accordingly. However, since we submitted our work as "Software Submission", it is expected to be more concise compared to a research article (https://journals.plos.org/ploscompbiol/s/submission-guidelines#loc-software-submissions)

Major points

1. Overall, the authors should make a better case for this tool. As they state, there are existing tools for doing such alignments. For instance, STAlign was recently published (PMID: 38065970) and landmark-based affine transformation has been used for some time. The authors cite existing approaches, but they don't compare the performance of their tool to the state-of-the-art, or discuss in any depth the unique use cases for BoReMi-based alignment beyond the need for generating manual ground truth. At a minimum, this should be argued in more detail. Even better, the authors should compare performance to existing approaches and possibly even show an instance of how BoReMi alignment can be used to solve a problem or address a question inaccessible with existing approaches.

We now show an example where STalign (as an example for a state-of-the-art automated alignment tool) over-distorted the data possibly due to difficulties in setting landmarks as the sample (Slide-seq of mouse hippocampus) does not contain any outer border (Figure 5). BoReMi however, allows the display of internal structures making landmark matching much easier and it does not automatically apply distortions (if applicable these can be added by the user through the newly implemented non-linear transformation functionality in a controlled manner).

We do not see BoReMi as a competitor of automated alignment tools but rather as complementary to either enable the entire process (tricky cases, ground-truth generation), serve as initial “rough” aligner, or as subsequent “fine” aligner.

2. A single example of data integration with a single type of molecular data (MERFISH data) is given. This example is furthermore not at single-cell resolution and uses very structured image data (i.e., brain tissue, with alignment based on hippocampus structure). Does BoReMi work for single cells? For tissue with more homogenous structure (e.g., tumors)? Does it work for data types other than MERFISH? The manuscript and potential users would benefit from some more examples and a clearer assessment of cases where this approach is/is not likely to be beneficial.

We now show the application of BoReMi across a range of different methods and tissues. Specifically we use single-cell segmented MERFISH of whole mouse brain (Figure 2 and Figure 3), Slide-seq of the mouse hippocampus regions (Figure 5), and single-cell segmented MERFISH and Slide-seq (as serial sections) of a breast cancer metastasis biopsy with very little internal structure (Figure 4). With Slide-seq and MERFISH representing two very different spatio-molecular data types we hope to efficiently cover the range of available methods.

3. Importantly, there is no assessment of how well the manual alignment with BoReMi works, neither any sort of sanity check (e.g., do well studied cell types or tissue regions show the expected molecular profiles?), nor any quantitative assessment. The authors are proposing that alignments generated by BoReMi can be used as ground truth to quantitatively check automated alignment methods; isn’t it crucial then that the manual alignment is accurate? How would the authors assess this in their own work and how would a user of BoReMi be expected to do so?

Indeed quantitative assessments of alignment quality are desirable but they are inherently difficult to achieve directly for data-to-image registration without ground-truth. We propose to use manual alignments as ground-truth the same way manual segmentation has traditionally served as ground-truth when developing cell segmentation algorithms. This is based on the assumption that humans are very good at “flexible visual pattern recognition”. But humans are also very slow, expensive, and not quantitative, creating the need for automated tools.

Quantitative assessment of data-to-image registration is (to our knowledge) only possible by proxy: Either through “validation by region composition”, where the region annotation from the image is transferred to the data and expected region characteristics are then inferred from the data (as suggested in this reviewer comment) - or through molecular similarity between aligned data generated from serial sections, which has the potential caveat of variation in the Z direction.

Unfortunately, in this version BoReMi does not yet allow region annotation transfer making the suggested “validation by region composition” approach not directly applicable since using an

external region annotation transfer method would make the benchmark dependent on another tool. Instead, we used the second possibility and quantitatively assessed the quality of our registration by spatial correlation of expression between serial sections and by calculating the overlap between data and HE (Figure 4). For this purpose we use a “hard case” of cancer tissue (breast cancer metastasis) with little internal structure (as suggested in reviewer comment #2), for which multiple serial sections are available in addition to a matching HE image.

Additionally, we show the level of precision that can be achieved with BoReMi by single-cell matching between cell-segmented MERFISH data and a DAPI image from the same tissue section (Figure 3b), which admittedly is not quantitative but should still add some confidence.

4. The manuscript states (lines 228-229) that cell type assignments or clusters are optional, but this did not seem to be the case when we tested the tool. These annotations appear mandatory, meaning that preprocessing of the molecular data is necessary to use BoReMi. What are the requirements for this preprocessing? There is no information provided on input file format specifications for the molecular data (e.g., csv with cell ids, center cell coordinates, other?); any requirements should be described in detail. If this type of preprocessing or annotation is not mandatory, the authors should then show how the alignment works without it.

We apologize for the confusion. Annotations are indeed optional, however, it is necessary to set the respective variable (“clusters_column_names”) in the notebook to “none” such that BoReMi does not expect annotation information. We have now made this parameter and its possible states more obvious by adding a comment.

5. How does BoReMi deal with any need for non-linear transformations, or combinations of linear and non-linear transformations? How does it deal with things like tissue tears or folds?

We have now implemented the possibility to perform non-linear transformations and demonstrate its use to fix a small tear in Figure 3A. To streamline the process, we require the user to first perform all required linear-transformations and then switch BoReMi to “non-linear” mode which allows users to click and drag individual data points together with their surrounding data points in an elastic manner to maintain neighborship relations. We note that this feature can still be improved by introducing more parameters for a more individualized behavior, releasing it as “beta” at this state.

6. From a user perspective, it would be extremely practical to have a reset/undo button in BoReMi, for instance if one makes a mistake or decides to try a different strategy during alignment. At present it seems one must re-do the full alignment process in such cases.
We have now implemented an undo/redo functionality for the linear transformations and the option to reset to the state after linear alignment for the non-linear transformations

7. In the README on GitHub, inexperienced users would benefit from a more detailed explanation of how to create the conda-environment (e.g. conda create -n) and how to install respective package from the .txt (e.g. conda install --file requirements.txt). In general, the more tutorial-like information that can be generated for users, the better.

We have now updated the README.md accordingly.

Minor points

8. On lines 250-252, the manuscript refers to a new image having to be selected. This is confusing. Isn't it the case that there is a single image (i.e., the consecutive section) that needs to be aligned to the molecular (MERFISH) data? Why should one be able to select one out of many images?

We apologize for the confusion. In this example we attempt the challenging task to align data to an HE image of the Allen Brain Atlas. This should demonstrate a use-case where no HE image has been generated together with molecular data. Since here, data and HE do not come from serial sections it is part of the alignment problem to select the best-fitting image out of a data-base. With the possibility to switch between different images, BoReMi makes this easier. We have modified the text to better represent this scenario.

9. It was not possible to reproduce the results from the paper using the settings shown in Figure 2. Perhaps these settings were not intended for this purpose, but we suggest that the values shown in the figure should then be corrected, or the correct settings given somewhere clearly in the paper (if the tool is indeed deterministic).

We have now re-made the figure such that values shown reproduce the alignment. The linear transformations are indeed deterministic. We also make the registration parameters as output by BoReMi for reference available on github.

10. Cluster selection no longer works once regions are selected with the toolbar (e.g. box selection) – is this a bug in the software?

Thank you for making us aware of this bug - the box selection widget is not yet integrated properly. We removed it for the time being.

11. The step sizes used for adjusting the data point size and the x/y coordinates are very small. Is there a reason for this? Is it tailored for single-cell matching? That would make it all the more compelling to show such an example.

The step sizes for adjusting the data point position are indeed small, now set at increments of ±0.5 to make the use of the buttons more efficient. Previously the step size was 0.001 - this precision can still be achieved if the value is directly entered. This high precision is intentional to facilitate fine-tuned adjustments especially when zooming in during applications such as single-cell matching. We now demonstrate this capability in Figure 3B by aligning the MERFISH data to a DAPI image of the same tissue section.

12. It may be useful to include a small example dataset directly in the GitHub repository.

The GitHub repository now contains a sample MERFISH dataset and HE/ DAPI images, along with their sources. Binder utilizes this data to demonstrate the application of BoReMi. The README.md now provides links to the respective repository folders and the original data sources.

13. The authors could consider integrating this with popular python-based image viewers such as napari (as a plugin).

We plan to enable this functionality in future versions, which will indeed enhance the applicability of BoReMi.

14. It would be helpful to mention minimal computational requirements for smooth running of BoReMi if any (e.g., memory use). Also, we also found no information on whether the software would be frozen or continually updated; it would be good to know in regard to software unit testing and validation checks.

We now mention computational requirements in the manuscript and on GitHub: "BoReMi has modest resource requirements: On a Surface Pro 7 tablet (Intel Core i5-1035G4, 16GB memory) it runs smoothly and opens the Merfish mouse brain dataset with 83k cells (subsampled to 10k cells for visualization) and images with 100 MP in about two minutes for PNG images and in about 1 minute for JPEG images. Downsampling the number of displayed data points to 10k, while all data points are being processed, ensures lag-free interaction even with modest computational resources." Versions will be frozen and tagged such that older versions remain available through GitHub.
